# DIAPH1 Promotes Laryngeal Squamous Cell Carcinoma Progression Through Cell Cycle Regulation

**DOI:** 10.3389/fonc.2021.716876

**Published:** 2021-09-22

**Authors:** Jiechao Yang, Qiang Huang, Yang Guo, Zheqiang Wei, Liang Zhou, Hui Chen

**Affiliations:** ^1^ Department of Otorhinolaryngology-Head and Neck Surgery, Wuxi No. 2 People’s Hospital, Affiliated Wuxi Clinical College of Nangtong University, Wuxi, China; ^2^ Department of Otorhinolaryngology-Head and Neck Surgery, Eye, Ear, Nose, and Throat Hospital, Fudan University, Shanghai, China; ^3^ Department of Pathology, Affiliated Hospital of Jiangnan University, Wuxi, China

**Keywords:** LSCC, DIAPH1, cell cycle regulation, G1/S progression, mitosis

## Abstract

The diaphanous related formin 1 (DIAPH1) protein is involved in the regulation of dynamic cytoskeleton reorganization, which is closely related to mitosis and the cell cycle. Cell cycle disorders are generally regarded as important underlying causes of many cancers. In the current study, we have revealed that DIAPH1 expression is an independent prognostic factor for overall survival in patients with laryngeal squamous cell carcinoma (LSCC) and that DIAPH1 promotes colony formation, cell proliferation, and G1/S progression in LSCC cells. Additionally, DIAPH1 promotes growth of AMC-HN-8 LSCC-derived tumors *in vivo*. In this study, RNA-sequencing analysis revealed that DIAPH1 knockdown led to changes in the expression of genes associated with signaling during the cell cycle. Using western blot analyses, we further demonstrated that DIAPH1 knockdown resulted in upregulation of p21^Waf1/Cip1^, p19^Ink4d^, p27^Kip1^, and p16^Ink4a^ and downregulation of cyclinA2, cyclinD1, CDK2, CDK4, and CDK6. These results suggest that DIAPH1 influences the expression of genes in several signaling pathways and promotes LSCC progression by regulating the cell cycle.

## Introduction

Laryngeal squamous cell carcinoma (LSCC), accounting for more than 95% of all laryngeal carcinomas (1), is a common type of malignant tumor in the head and neck region. Based on cancer statistics from the National Central Cancer Registry of China, it was estimated that 26,400 new cases of laryngeal carcinoma and 14,500 cancer deaths were due to laryngeal carcinoma in China in 2015 (2). Similarly, Siegel previously reported of the approximately 12,410 cases diagnosed with laryngeal carcinoma in the United States in 2019, 3,760 would lead to deaths (3). Development of diagnostic and treatment technologies have improved the 5-year survival rate of most cancers, but laryngeal cancer, for which the 5-year survival rate has decreased in the past 40 years (4), is an unfortunate exception. Hence, the search for new diagnostic and therapeutic targets for laryngeal cancer, especially for LSCC, has become more pressing.

The initiation and development of tumors are closely related to cell cycle disorders. To stabilize the genome, mammalian cells have a complicated system to respond to DNA damage from a variety of injuries that may occur during the course of DNA replication in the cell cycle. Loss of cell cycle control due to failure of DNA damage and repair machinery can result in an increased rate of mutation and continued division and proliferation of the cells. This uncontrolled growth may lead to development of various types of tumors, such as breast cancer (5).

So far, many research literatures have confirmed that cell cycle regulation get involved in the pathogenesis of laryngeal cancer. For example, overexpression of cyclin-D1 can be used as a marker to predict relapse in patients with LSCC after primary curative resection (6); circMYLK/miR-195/cyclin D1 regulatory axis could affect the proliferation and cell cycle progression of LSCC cells (7); in relation to the simultaneous expression of p27 protein and cyclin D1, the patients with a cyclin D1+/p27- phenotype had the poorest disease-free and overall survival rates (8).

The cell cycle of mammalian cell is a course of mitosis, accompanied by dynamic reorganization of the cytoskeleton. Previous studies have shown that formin activity interferes with initiation of DNA replication (9) and that the formin protein is critical to cell cycle-specific nucleosome organization (10). Using 36 paired LSCC and matching adjacent non-tumor tissues, we previously showed that expression of the protein diaphanous related formin 1 (DIAPH1, also termed mDia1), a member of the formin family, is significantly upregulated in LSCC tissues. Furthermore, in LSCC cells, DIAPH1 knockdown significantly increased levels of ataxia-telangiectasia- and Rad3-related (ATR) proteins (11). ATR is an indispensable kinase for human and mouse DNA replication, affecting cell cycle transformation, DNA damage responses, and apoptosis. Activated ATR can stabilize and repair replication forks and prevent premature mitosis (12). Thus we hypothesized that DIAPH1 participates in progression of cancers by virtue of its regulatory role in the cell cycle and sought to elicit the specific regulatory interactions mediated by DIAPH1 that are associated with development and progression of LSCC.

## Materials and Methods

### Patient Details and Tissue Sample Collection

Paraffin-embedded samples of LSCC tissues from 126 patients with LSCC who underwent total or partial laryngectomy at the Department of Otorhinolaryngology-Head and Neck Surgery between February 2009 and November 2010 were obtained from the Pathology Department of the Eye, Ear, Nose and Throat Hospital of Fudan University. Patients selected for this study had not received chemoradiotherapy or other biological therapies before surgery and all provided written informed consent while hospitalized. Approval for this study was obtained from the Ethical Committees of the Eye, Ear, Nose and Throat Hospital. TNM and clinical classification of all patients were performed according to the American Joint Committee on Cancer guidelines, and the clinicopathological characteristics and follow-up details of all patients were collected from telephone records, outpatient records, and patient charts. Overall survival (OS) was calculated from the date of the first surgery to the date of death or last follow-up. Follow-up was continued through April 2016. **Table 1** shows the demographics and clinical characteristics of all 126 LSCC patients. The mean age of the patient cohort was 61 years, and the patients were divided into “older” (≥61 years) and “younger” (<61 years) groups for analyses.

**Table 1:** The demographics and clinical characteristics of 126 LSCC patients.

Characteristics	No. of patients (%)
**Age** (<61/≥61 years)	65 (51.6%) /61 (48.4%)
**Gender** (Female/male)	2 (1.6% ) /124 (98.4%)
**Smoking history** (No/Yes)	62 (49.2%) /64 (50.8%)
**Drinking history** (No/Yes)	96 (76.2%) /30 (23.8%)
**Tumor subsite**	
Supraglottic/Glottic/subglottic	42 (33.3%) /78 (61.9%) /6 (4.8%)
**Tumor stage**	
T1/T2	19 (15.1%) /62 (49.2%)
T3/T4	38 (30.2%) /7 (5.6%)
**Lymph node stage**	
N0	102 (81.0%)
N+ (N1& N2& N3)	24 (19.0%)
**Clinical stage**	
I/II	19 (15.1%) /48 (38.1%)
III/IV	37 (29.4%) /22 (17.5%)
**Differentiation**	
Well/moderate/poor	48 (38.1%) /73 (57.9%) /5 (4.0%)
**DIAPH1 expression**	
Low/High	78 (61.9%) /48 (38.1%)

### Immunohistochemistry

Immunohistochemistry (IHC) was performed on paraffin sections as previously described, and an immunohistochemical score (IHS) value was used to estimate the level of DIAPH1 expression (11). The patients were divided into high (≥6) and low (<6) expression groups based on the median DIAPH1 IHS value.

### Cell Lines and Cell Culture

We used two LSCC cell lines, AMC-HN-8 and FD-LSC-1 cells, to perform the related experiments. The origin and culture of the cells were the same as described previously (11).

### Construction of DIAPH1 RNA Interference Vectors and Lentiviral Infection

**Figure 1:**
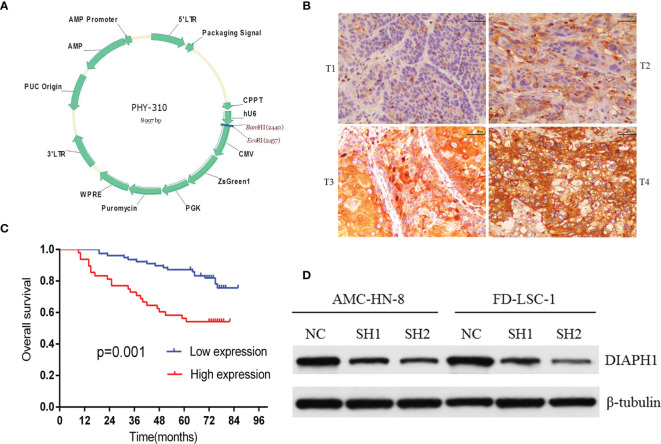
The relationship between DIAPH1 expression and clinical samples and DIAPH1 knockdown efficiency in cells. **(A)** The lentivirus vector atlas used in DIAPH1 knockdown by shRNA lentivirus. **(B)** Immunohistochemistry analysis of DIAPH1 expression of laryngeal squamous cell carcinoma at different tumor stages (T1-T4, scale bar 50 μm). **(C)** Kaplan–Meier curves for overall survival stratified according to DIAPH1 protein expression level. **(D)** Western blot analysis of DIAPH1 protein expression in DIAPH1 knockdown (SH) cells and the negative control (NC) cells.

Stable DIAPH1 knockdown (SH1, SH2) and negative control (NC) cell lines were obtained as previously described (11). Briefly, using two short hairpin RNA (shRNA) sequences targeting the DIAPH1 gene, we successfully infected AMC-HN-8 and FD-LSC-1 cells with the recombinant DIAPH1-shRNA lentivirus (SH1, SH2) and the negative control (NC) lentivirus. The two shRNA sequences were: SH1, GGACAAAGGTGAAGGAGGA; SH2, GGAGAAATCTGAAGCCAAA. **Figure 1A** shows the lentivirus vector atlas.

### Colony Formation Assay

Colony formation assays were performed for the above-mentioned three groups of AMC-HN-8 cell lines, which were seeded into six-well plates at a density of 1000 cells/well. The cells were continuously incubated for 10 days under culture conditions described above. All colonies were fixed with paraformaldehyde (1 mL/well, Dingguo Changsheng Biotechnology, Beijing, China) followed by crystal violet staining (500 µL/well, Beyotime, Shanghai, China). Finally, the colonies that contained more than 50 cells were counted using an inverted light microscope (Nikon ECLIPSE Ti, Japan). The same method was applied to the two DIAPH1 RNA-interference groups and one NC group of FD-LSC-1 cells. For the FD-LSC-1 cells, the planting density was reduced to 800 cells/well and the incubation was extended to 11 days.

### Proliferation Assay

The Cell CountingKit-8 (CCK-8, Dojindo, Kumamoto, Japan) was used to assay the proliferation of AMC-HN-8 cells. The two DIAPH1 RNA-interference groups and one NC group of AMC-HN-8 cell lines were seeded into 96-well plates at a density of 2000 cells/well and cultured as described above. The proliferation assay was performed according to the manufacturer’s instructions; briefly, the CCK-8 (10 µL/well) and culture medium (100µL/well) were added into the wells at 24, 48, 72, and 96 h after planting. We measured absorbance at 450 nm using the Synergy H1 Hybrid Microplate Reader (Bio-Tek, USA) after incubating for 2 h. The same method was used to assay the three groups of FD-LSC-1 cell lines except that a planting density of 3000 cells/well was used and the CCK-8 and culture medium were added into the wells at 6, 24, 48, 72, and 96 h after planting.

### Cell Cycle Assay

The three groups of AMC-HN-8 or FD-LSC-1 cell lines were simultaneously planted into 10 cm cell culture dishes and incubated routinely for 10 h. The cells were then serum-starved for 24 h before routine culture with FBS for another 24 h. The cells were collected and enumerated with a hemocytometer to ensure that there were 1–2×10^6^ cells/tube. Cold PBS was used to wash the cells twice, and the centrifugated cells were resuspended in 300 µL precooled PBS. Chilled 75% ethanol was then used to fix the cells overnight. After centrifugation and washing with cold PBS, the cells were resuspended in 0.5 ml PI/RNase staining buffer (BD PharMingen, CA, USA) and incubated for 15 min at RT in the dark. Finally, the cells were analyzed by flow cytometry (BD FACSCalibur, USA) within 1 h.

### *In Vivo* Tumorigenicity Assay

Eighteen 4-week old male NOD/SCID mice were fed in the required environment and divided randomly into three equal groups. Two DIAPH1 RNA-interference groups and one NC group of AMC-HN-8 cell lines were harvested and washed with RPMI-1640. Three groups of cells were mixed with RPMI-1640 and the concentration of the cell suspension was adjusted to 2 ×10^7^ cells/ml. The cell suspension (200μl) was subcutaneously injected into the left armpit of the mice in each group. The mice were observed every day. On the sixth day after injection, the long diameters (L) and the short diameters (S) of the tumors were measured using a Vernier caliper. Subsequently, the measurements were performed every three days until the twenty-first day. The mice were sacrificed by cervical dislocation 21 d after injection and the tumors were completely taken out and weighed. The growth curve of the tumor was evaluated by the volume (V) of the tumor, which was calculated using the formula, V= S^2^ × L.

### RNA-Sequencing Analysis and Enrichment Analysis for AMC-HN-8 Cell Lines

Two DIAPH1 RNA-interference groups and one NC group of AMC-HN-8 cell lines were harvested and total RNA was extracted from the cells using the mirVana™ miRNA Isolation Kit (Ambion-1561, Invitrogen™, USA) according to the manufacturer’s protocol. RNA was quantified using a NanoDrop 2000c spectrophotometer (Thermo Scientific Inc., Waltham, MA, USA) and RNA integrity was evaluated using an Agilent 2100 Bioanalyzer (Agilent Technologies, Santa Clara, CA, USA). Samples with RNA Integrity Number (RIN) ≥ 7 were used for subsequent analyses. The libraries were constructed using a TruSeq Stranded mRNA LTSample Prep Kit (Illumina, San Diego, CA, USA) according to the manufacturer’s instructions. The libraries were sequenced on an Illumina sequencing platform (HiSeq™ 2500) and 150 bp paired-end reads were generated.

Raw data were processed to obtain clean reads and the clean reads were mapped to the reference genome using hisat2. The FPKM value of each gene was calculated using cufflinks, and the read counts of each gene were obtained by htseq-count. Differentially expressed genes (DEGs) were identified using the DESeq (2012) function estimateSizeFactors and nbinomTest. A P-value < 0.05 was set as the threshold for significantly differential expression. Hierarchical cluster analysis of DEGs was performed to explore gene expression patterns. Gene Ontology (GO) enrichment and Kyoto Encyclopedia of Genes and Genomes (KEGG) pathway enrichment analysis of DEGs were performed using R based on the hypergeometric distribution.

### Western Blot Analysis

Proteins were harvested from the three groups of AMC-HN-8 or FD-LSC-1 cell lines and use for western blotting, as previously described (11). The reagents and instruments used were as follows: RIPA protein extraction reagent (Beyotime, Shanghai, China), protease inhibitor cocktail (Weiao Biaotec, Shanghai, China), BCA protein assay (Beyotime), polyvinylidene fluoride membranes (PVDF, Millipore Corporation, Bedford, MA, USA), and TBST (Sangon Biotech, Shanghai, China). In addition, the primary antibodies used were anti-DIAPH1 (1:1000; Abcam, UK, cat. no. ab129167), anti-p27^Kip1^ (1:1000, Cell Signaling Technology [CST], USA, cat. no. 3686), anti-p16^Ink4a^ (1:1000, Abcam, cat. no. ab81278), anti- p21^Waf1/Cip1^ [1:500, Santa Cruz Biotechnology (Santa), USA, cat. no. sc-817], anti-p19^Ink4d^ (1:500, Santa, cat. no. sc-1665), anti-cyclinD1(1:1000, CST, cat. no. 92G2), anti-cyclinA2(1:2500, Abcam, cat. no. 32386), anti-cyclin-dependent kinase 2 (anti-CDK2, 1:500; Santa, cat. no. sc-6248); anti-CDK4 (1:3000, CST, cat. no. 12790); anti-CDK6 (1:3000, Abclonal, Wuhan, China, cat. no. A0705), anti-GAPDH (1:1000, Abcam, cat. no. AC002), and β-tubulin (1:5000, Servicebio, Wuhan, China, cat. no. GB13017-2). The PVDF membranes were incubated with primary antibodies overnight at 4°C and then with horseradish peroxidase-conjugated secondary antibodies (1:2000, Jackson ImmunoResearch, USA) for 2 h at room temperature. Finally, enhanced chemiluminescence reagent (ECL Kit, Beyotime) and autoradiography were used to visualize the protein bands (Carestream Health, Canada).

### Statistical Analysis

SPSS 22.0 (SPSS Inc., Chicago, IL) and GraphPad Prism 5 software (San Diego, CA) were used to perform statistical analyses. Except for bioinformatics analyses, all data are presented as the percentage (%), mean ± standard deviation, hazard ratio (HR), and 95% confidence interval (95% CI). A p-value < 0.05 was considered statistically significant. Differences between groups were evaluated using the appropriate method selected from the following methods: the Mann-Whitney U test, the Student’s t-test, and the Kruskal-Wallis H test. Survival curves were calculated using the Kaplan–Meier method, and differences were compared using the log-rank test.

## Results

### Relationship Between DIAPH1 Expression and LSCC Clinicopathological Features

IHC analysis of 126 patients showed that DIAPH1 protein expression was significantly upregulated in patients with high T stage, lymph node metastasis, later clinical-stage, or supraglottic or subglottic LSCC (**Table 2**, **Figure 1B**). Age, T stage, clinical stage, and DIAPH1 expression were significantly associated with OS (**Table 3**, all p<0.001, **Figure 1C**). Moreover, age and DIAPH1 expression were significant independent prognostic factors for OS (**Table 4**, p=0.025 and 0.001, respectively).

**Table 2:** The relationship between DIAPH1 expression and clinical pathological features in 126 LSCC patients (*p<0.01).

Clinical pathological features	HIS Mean Rank	P value
**Age (years)**		0.783
<61/≥61	64.35/62.59	
**Gender**		0.897
Female/male	66.75/63.45	
**Smoking history**		0.575
No/Yes	65.32/61.73	
**Drinking history**		0.612
No/Yes	64.41/60.60	
**Tumor subsite**		<0.001*
Supraglottic/Glottic/subglottic	79.19/52.57/95.75	
**Tumor stage**		<0.001*
T1/T2/T3/T4	38.34/49.54/91.14/105.36	
**Lymph node stage**		0.002*
N0/N+ (N1& N2& N3)	58.79/83.50	
**Clinical stage**		<0.001*
I/II/III/IV	38.34/41.53/86.66/94.20	
**Differentiation**		0.579
Well/moderate/poor	59.34/66.30/62.50	

**Table 3:** Univariate analysis of the demographics and clinical characteristics related to overall survival (*p<0.05).

Variables	Hazard ratio	95% Confidence interval	P value
**Age (years)**		1.03-3.65	0.04*
<61/≥61	1.00/1.94		
**Gender**		0.06-6.94	0.73
Female/male	1.00/0.66		
**Smoking history**		0.50-1.75	0.83
No/Yes	1.00/0.93		
**Drinking history**		0.56-2.53	0.65
No/Yes	1.00/1.19		
**Tumor subsite**		0.27-1.08	0.08
Supraglottic/Glottic & subglottic	1.00/0.54		
**Tumor stage**		1.78-7.06	<0.01*
T1-T2/T3-T4	1.00/3.55		
**Lymph node stage**		0.89-5.03	0.09
N0/N+ (N1& N2& N3)	1.00/2.12		
**Clinical stage**		1.37-4.94	<0.01*
I-II/III-IV	1.00/2.60		
**Differentiation**		0.55-2.02	0.87
Well/Moderate & poor	1.00/1.05		
**DIAPH1 expression**		1.55-5.98	<0.01*
Low/High	1.00/3.05		

**Table 4:** Multivariate cox proportional hazard regression for overall survival (*p<0.05).

Variables	Group	Hazard ratio	95% Confidence interval	P value
**Age (years)**	≥61 *vs* <61	2.07	1.08-3.96	0.025*
**Clinical stage**	III-IV *vs* I-II	1.59	0.69-3.67	0.275
**DIAPH1 expression**	High *vs* Low	2.86	1.51-5.41	0.001*

### Construction of DIAPH1-Knockdown Cells

AMC-HN-8 and FD-LSC-1 cells were infected with shRNA sequences targeting the DIAPH1 gene; stable DIAPH1 knockdown (SH1, SH2) and NC cell lines were successfully constructed. Western blot analysis verified the knockdown efficiency of DIAPH1 in AMC-HN-8 and FD-LSC-1 cells (**Figure 1D**).

### DIAPH1 Promotes LSCC Cell Colony Formation and Proliferation

**Figure 2:**
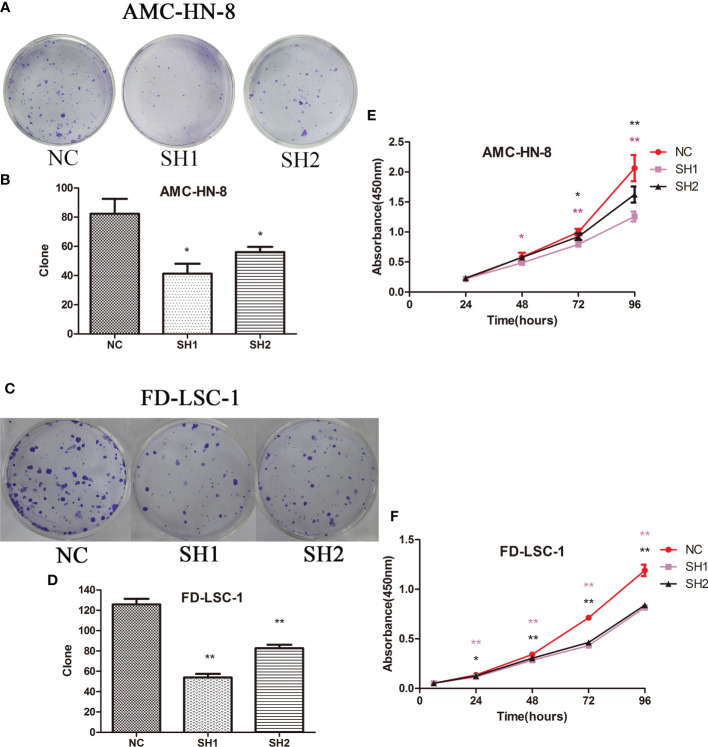
Colony formation assay and CCK-8 assay of DIAPH1 knockdown (SH) cells and the negative control (NC) cells. **(A, C)** Representative graphs of colony formation of the indicated cells. **(B, D)** Comparison of the clonogenicity of the indicated cells between DIAPH1 SH1, SH2 and NC groups. **(E, F)** Cell proliferation was assessed in DIAPH1 SH1, SH2 and NC groups using CCK8 assays ^∗^p < 0 05, ^∗∗^p < 0 01.

There were significantly fewer colonies formed by SH1 and SH2 AMC-HN-8 cells (41.33 ± 11.59, P=0.021 and 54.33 ± 8.14, P=0.049, respectively) than were formed by DIAPH1-NC cells (83.33 ± 15.95; **Figures 2A, B**). The same trend was observed in the FD-LSC-1 cells (NC, 125.67 ± 9.61; SH1, 54.00 ± 6.08, P=0.000; SH2, 82.67 ± 5.86, P=0.003; **Figures 2C, D**). The CCK-8 assay showed that AMC-HN-8 DIAPH1-knockdown cells had lower optical density than controls, with significant differences in optical density emerging 48 h after planting (**Figure 2E**). The same phenomenon was observed in FD-LSC-1 DIAPH1-knockdown cells (SH1 and SH2), except that significant differences emerged 24 h after planting (**Figure 2F**). Together, these results indicating that DIAPH1-knockdown suppressed formation of colonies by single cells *in vitro* and cell proliferation *in vitro*.

### DIAPH1 Promotes G1/S Progression in LSCC Cells

**Figure 3:**
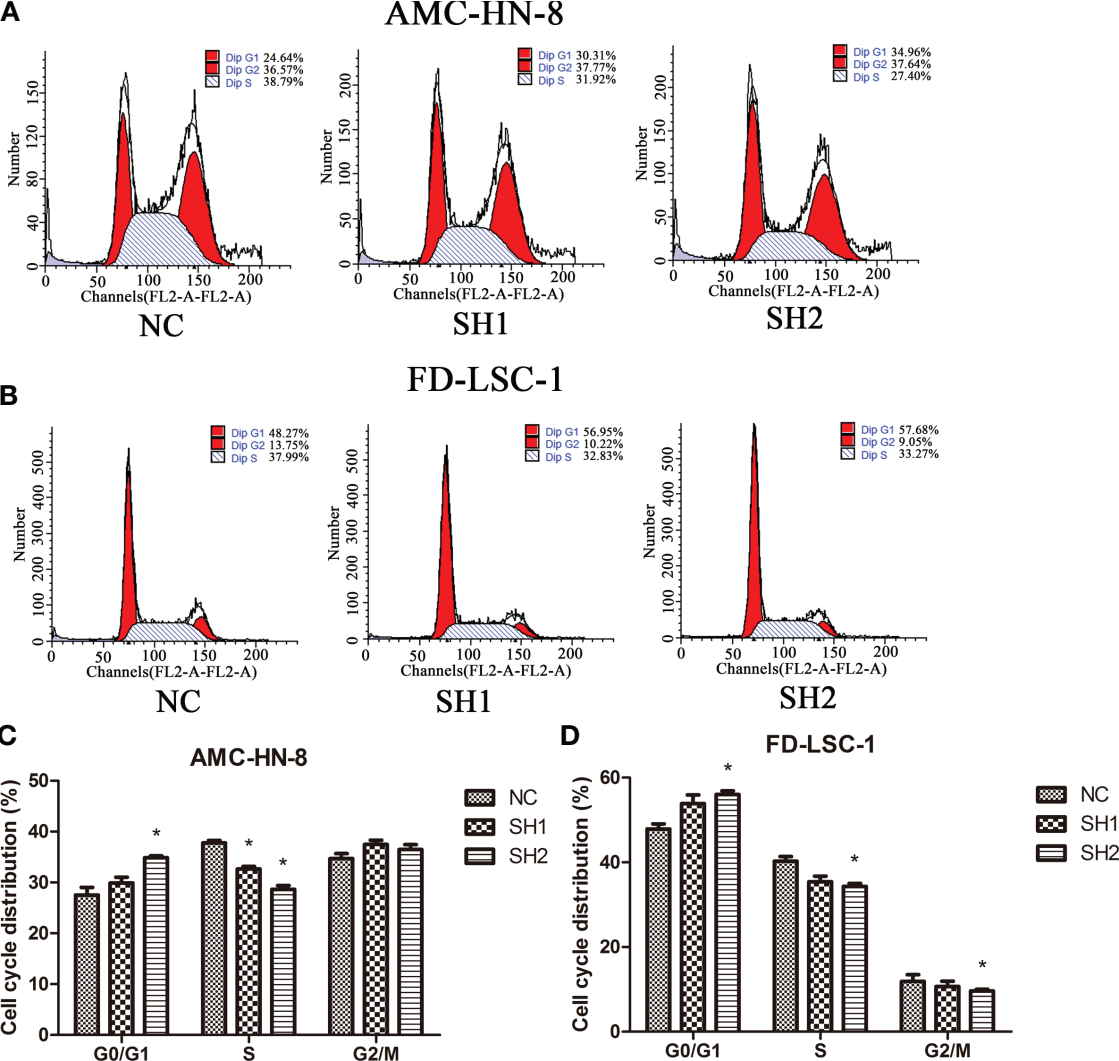
Cell cycle distribution of DIAPH1 knockdown (SH) and the negative control (NC) as detected by flow cytometric analysis. **(A, B)** Representative graphs of cell cycle distribution in AMC-HN-8 and FD-LSC-1 cells by flow cytometric analysis. **(C, D)** Comparison of the proportional distribution of cell cycle between three groups. ^∗^p < 0 05.

The effect of DIAPH1 knockdown on the cell cycle in AMC-HN-8 cells was measured by flow cytometry. In AMC-HN-8 cells, the number of S phase NC cells (37.77 ± 0.88%) was significantly higher than the number of S phase of SH1 and SH2 cells (32.65 ± 0.85%, P=0.002 and 28.66 ± 1.28%, P=0.001, respectively; **Figures 3A, C**). Meanwhile, the number of G0/G1 phase NC cells (27.53 ± 2.58%) was lower than the number of G0/G1 phase SH1 and SH2 cells (29.88 ± 2.01%, P=0.281 and 34.86 ± 0.62%, P=0.009, respectively), though the difference was only statistically significant in SH2 cells. Similarly, a larger number of G0/G1 phase FD-LSC-1 SH1 and SH2 cells (53.88 ± 3.55%, P=0.065 and 56.04 ± 1.46%, P=0.005, respectively) than FD-LSC-1 NC cells, (G0/G1, 47.87 ± 2.09%; S, 40.26 ± 1.98%; G2/M, 11.88 ± 2.81%; **Figures 3B, D**) were observed, and fewer S and G2/M phase FD-LSC-1 SH1 and SH2 cells (S, 35.43 ± 2.27%, P=0.050; G2/M, 10.69 ± 2.14%, P=0.590 and S, 34.33 ± 1.17%, P=0.011; G2/M, 9.64 ± 0.65%, P=0.299, respectively) cells were present. These results suggest that DIAPH1 promotes G1/S progression in LSCC cells *in vitro*.

### DIAPH1 Promotes the Growth of AMC-HN-8 LSCC-Derived Tumors *In Vivo*

**Figure 4:**
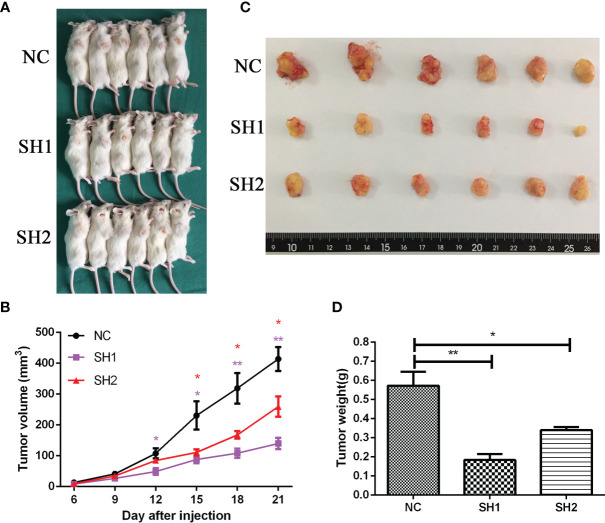
DIAPH1 promotes the growth of AMC-HN-8 LSCC-derived tumors in NOD/SCID mice. **(A)** The transplanted tumor models were established by DIAPH1 knockdown (SH) cells and the negative control (NC) cells. **(B)** Tumor growth assay of three groups *in vivo*. **(C)** The tumor images 21 days after implantation. **(D)** Comparison of average weight of the tumor 21 days after implantation between three groups. ^∗^p < 0 05, ^∗∗^p < 0 01.

To further explore the *in vivo* function of DIAPH1, 18 male NOD/SCID mice transplanted with AMC-HN-8 cells were used to establish a transplanted tumor model (**Figure 4A**). As shown in **Figure 4B**, significantly more rapid tumor growth occurred in the NC group than in the two DIAPH1-knockdown groups. The tumor weight of the NC group was significantly higher than that of the two DIAPH1-knockdown groups 21 d after transplant (**Figures 4C, D**), suggesting that DIAPH1-knockdown suppresses tumor growth in NOD/SCID mice.

### DIAPH1-knockdown Alters Expression of Downstream Signaling Pathways

**Figure 5:**
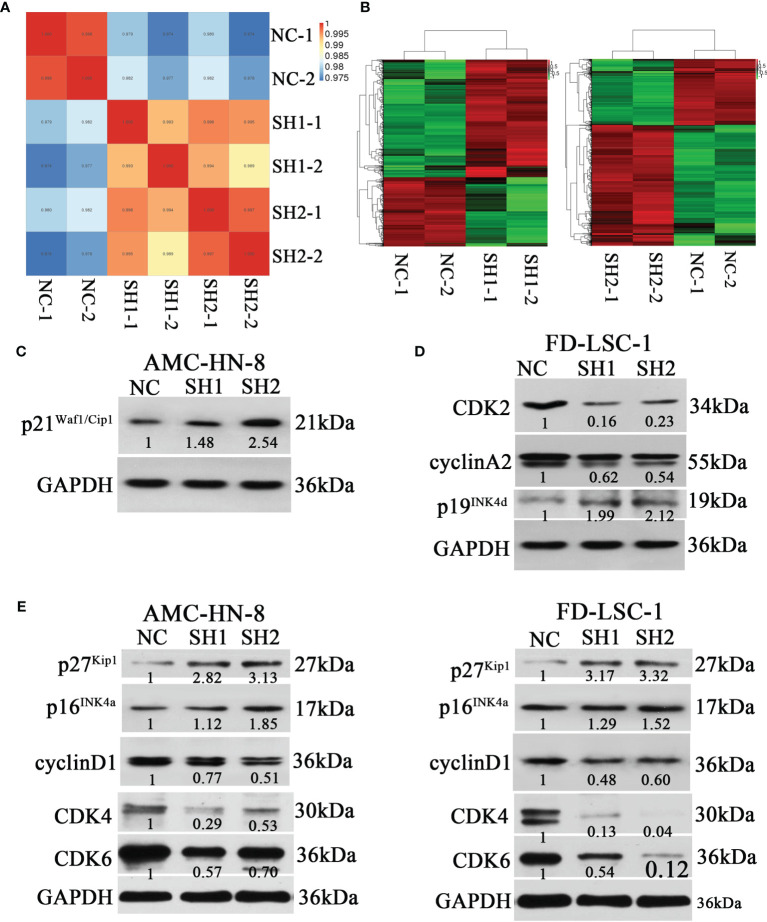
Differences of the expression of cell cycle-related proteins between DIAPH1 knockdown (SH) cells and the negative control (NC) cells. **(A)** Intersample correlation test of AMC-HN-8 cells by RNA-sequencing analysis on the Illumina sequencing platform. **(B)** Results of cluster analysis of differentially expressed genes in AMC-HN-8 cells. **(C–E)** Western blot analysis of the expression of cell cycle-related proteins in AMC-HN-8 and FD-LSC-1 cells. Numbers labeled under the bands were the relative expression and the relative expression of NC cells was set as 1.

To further explore the mechanism of DIAPH1, we performed RNA-sequencing of AMC-HN-8 NC, SH1, and SH2 cells. **Figures 5A, B** show the inter-sample correlation test and the DEG cluster analysis, respectively. GO enrichment and KEGG pathway enrichment analysis showed that DIAPH1 knockdown led to changes in many downstream signaling pathways, and signaling pathways associated with the cell cycle were critical (NC/SH1, p =5.59× 10^−5^; NC/SH2, p =6.02× 10^−5^).

Based on results from the RNA-sequencing analysis, we performed western blotting for cell cycle-related proteins. DIAPH1 knockdown resulted in an upregulation of p21^Waf1/Cip1^ (**Figure 5C**) in AMC-HN-8 cells and upregulation of p19^Ink4d^ and downregulation of cyclinA2 and CDK2 in FD-LSC-1 (**Figure 5D**). In addition, DIAPH1 knockdown resulted in upregulation of p27^Kip1^ and p16^Ink4a^ and downregulation of cyclinD1, CDK4, and CDK6 in both AMC-HN-8 and FD-LSC-1 cells (**Figure 5E**).

## Discussion

DIAPH1 belongs to a class of actin regulatory proteins and has been demonstrated to effectively regulate actin polymerization and microtubule stabilization (13, 14). It is regarded as an important factor in regulating physiological state, shape function, and pathological mechanisms of cells (15, 16). Abnormal expression of DIAPH1 can lead to abnormalities in stress fibers, pseudopodia, and microtubules of tumor cells, which influences proliferation, adhesion, and migration of tumor cells, potentially changing the course of the tumor (16, 17).

DIAPH1 has been reported to be involved in the progression of many types of tumors, such as breast cancer (18), colorectal cancer (19), and malignant glioma (20). In our previous research, we performed bioinformatics analysis and the results showed that high expression of DIAPH1 was associated with poor OS in LSCC. Moreover, IHC analysis of 36 paired LSCC and matching adjacent nontumor tissues showed DIAPH1 protein expression was significantly upregulated in LSCC tissues (11). In order to investigate the function of DIAPH1 in LSCC, we carried out both clinical researches and cytobiological researches. In the present study, IHC analysis of DIAPH1 revealed significant increases in DIAPH1 expression in the presence of factors associated with severe illness and short OS, such as high T stage, lymph node metastasis, high clinical stage, and primary supraglottic or subglottic tumors. This suggested that high DIAPH1 expression was associated with poor prognosis, and subsequent survival analysis confirmed that high DIAPH1 expression is an independent risk factor for poor OS. To the best of our knowledge, this is the first report that DIAPH1 expression is associated with the OS of LSCC patients.

Due to the change of tumor characteristics and the expression of DIAPH1 caused by chemoradiotherapy, only the patients without chemoradiotherapy experiences were included. For T4 patients who had very large tumor size, they were recommended to have neoadjuvant chemoradiotherapy so as to get more possibilities to have surgeries later, therefore, the proportion of T4 patients was relatively low, comparing to T2 and T3 patients. In this study, we included the LSCC patients who received surgeries in our hospital from February 2009 to November 2010, and only the patients who had neoadjuvant chemoradiotherapy were excluded. We believed the group of included patients could represented the characteristics of LSCC and the results of this research could be popularized. In addition, if the postoperative pathology confirmed high risk factors like neck lymph nodes metastasis, positive margin and vascular invasion, adjuvant chemoradiotherapy would be performed according to National Comprehensive Cancer Network guideline, in order to prolong the survival time, and also to decrease the statistical bias.

In our analyses, older age was also an independent risk factor for poor OS. Similarly, Zhang et al. noted that older age was significantly associated with poor postoperative survival rate (21). The reason for the reduced OS is that older patients undergoing partial or total laryngectomy have poorer systemic status, lower immunity, and susceptibility to pulmonary infections due to direct exposure of the trachea to the outside.

Analyses of clinical samples show that DIAPH1 plays an important role in the process of LSCC. We successfully constructed stable DIAPH1 knockdown (SH1, SH2) and NC cell lines and investigated DIAPH1 function *in vivo* and *in vitro*. Previous studies reported that DIAPH1 promoted the proliferation of human U87 glioblastoma cells (20), melanoma cells (22), and liver cancer cells (23)*in vitro* and that DIAPH1 knockdown inhibited growth of U87 malignant glioma cell-derived tumors in nude mice *in vivo* (20). In the present study, both colony formation assays and CCK-8 assays demonstrated that DIAPH1 promotes proliferation of AMC-HN-8 and FD-LSC-1 cells *in vitro*. Furthermore, we found that DIAPH1 knockdown suppressed growth of AMC-HN-8 cell-derived tumors in NOD/SCID mice. Our results corroborate earlier research reports, providing further evidence that DIAPH1 promotes the progress of LSCC.

Interestingly, although DIAPH1 is upregulated in many types of tumors and promotes tumor cell proliferation, migration, invasion, progression *in vivo*, it also suppresses apoptosis (11, 18–20, 23–26), the relationship between DIAPH1 and survival is inconsistent (23, 25, 26). Tian et al. reported that DIAPH1 promoted growth of hepatocellular carcinoma cell lines and was an independent risk factor for OS, yet higher DIAPH1 expression predicted better prognosis in patients with HCC (23). For ovarian cancer patients, high DIAPH1 expression was associated with low differentiated tumors, while high DIAPH1 expression was associated with increased OS (25). In contrast, mDia1 increases migration and chemotaxis of leukemia cells, and mDia1 deficiency was found to inhibit leukemia progression and prolong survival of recipient mice in a leukemia transfer model (26). A colorectal cancer study demonstrated that SCIN knockdown significantly reduced DIAPH1 expression and SCIN served as an independent predictor of poor prognosis, which implies that high DIAPH1 expression may be associated with poor prognosis (24). In the current study, we showed that high DIAPH1expression is an independent risk factor for poor OS in patients with LSCC. Schiewek et al. suspected that an increased response to chemotherapeutics caused by high DIAPH1 levels may explain why elevated DIAPH1 expression was associated with increased OS in ovarian cancer patients (25). However, we hypothesized that different biological characteristics of tumors and differences in response to chemotherapeutics may result in an inconsistent relationship between DIAPH1 and survival. To evaluate this, we performed chemosensitivity testing with cisplatin and found that DIAPH1 did not affect the chemotherapy sensitivity of LSCC cells (data not shown).

Because the proliferation of LSCC cells depends on mitosis, we performed a cell cycle test and found that DIAPH1 promoted G1/S progression in LSCC cells. Furthermore, RNA-sequencing analysis, GO enrichment, and KEGG pathway enrichment analysis showed that the cell cycle-associated signaling pathway was a critical downstream pathway affected by DIAPH1. Cyclin, CDKs, and cyclin-dependent kinase inhibitor (CDKI) are the three major groups of cell cycle regulators. P21^Waf1/Cip1^ and p27^Kip1^, identified at almost the same time, belong to the Cip/Kip class of CDKI, and both cause G1 phase arrest by interacting with cyclin D/CDK4/CDK6. Moreover, p21^Waf1/Cip1^ is a downstream target of p53 and is induced by activated wild-type p53. P27^Kip1^ prevents cells from entering S phase by negatively regulating cyclin A/CDK2 (27–30). Mi et al. found coatomer subunit beta 2 knockdown increased the fraction of human prostate carcinoma cells in the G1 phase and decreased the fraction of cells in the S phase *via* the p21^Waf1/Cip1^/p27^Kip1^ and the cyclin D1/CDK2/CDK4 pathways (31). It has been reported that mDia1 advanced G1/S phase progression by increasing SKP2 expression to promote degradation of p27^Kip1^ (32, 33). P16^Ink4a^ and p19^Ink4d^ belong to the Ink4 class of CDKI and lead to G1 phase arrest by binding to CDK4/CDK6 to be isolated from cyclin D (29).

Our study showed that upregulation of p27^Kip1^and p16^Ink4a^ expression and downregulation of cyclinD1, CDK4, and CDK6 occurred in two different LSCC DIAPH1 knockdown cell lines, implying that DIAPH1 promoted G1/S phase progression by p27^Kip1^, p16^Ink4a^, and the cyclin D1/CDK4/CDK6 pathways. Furthermore, in AMC-HN-8 DIAPH1 knockdown cells, p21^Waf1/Cip1^ expression was increased, whereas in the FD-LSC-1 cells there was no significant change in p21^Waf1/Cip1^ expression. This is because the FD-LSC-1 cell line harbors a mutant p53, rather than wild-type p53, which can induce p21^Waf1/Cip1^ (34). This suggests that in AMC-HN-8 cells, the p53/p21^Waf1/Cip1^ pathway is also involved in cell cycle aberration caused by DIAPH1. In FD-LSC-1 DIAPH1 knockdown cells, we also observed upregulation of p19^Ink4d^ and downregulation of cyclin A2 and CDK2, which implies that DIAPH1 also regulated the cell cycle in FD-LSC-1 cells *via* the p19^Ink4d^/cyclin D1/CDK4/CDK6 and p27^Kip1^/cyclin A2/CDK2 pathways.

Therefore, our results demonstrate that DIAPH1 regulates the cell cycle of LSCC cells *via* several signaling pathways, including p27^Kip1^, p16^Ink4a^, p19^Ink4d^, cyclinD1/CDK4/CDK6, p53/p21^Waf1/Cip1^, and cyclinA2/CDK2 pathways.

The current study has some limitations. First, morphological changes related to dynamic cytoskeletal recombination were not studied. The process of mitosis is accompanied by drastic changes in the cytoskeleton. DIAPH1, a regulator of the cytoskeletal system, probably participates in cell cycle regulation by regulating the cytoskeleton system. Further research will be focused on the changes in the microstructure. Second, our study revealed that knockdown of DIAPH1 would influence cell cycle and associated with several protein expressions in various pathways, which indicated that DIAPH1 promoted LSCC progression by regulating the cell cycle *via* several signaling pathways. However, the specific target genes in particular pathways have not been proven. In our following research, we will perform more experiments to confirm the target relationship between genes and pathways and their interaction functions.

## Conclusions

In summary, we confirmed that high DIAPH1expression was an independent risk factor for poor OS in LSCC patients and that DIAPH1 promoted LSCC progression by regulating the cell cycle *via* several signaling pathways.

## Data Availability Statement

RNA Sequencing data presented in the study are deposited in the Figshare repository (https://figshare.com/articles/dataset/counts_anno_xlsx/16574234?file=30666056). The raw data supporting the conclusions of this article will be made available by the authors, without undue reservation.

## Ethics Statement

The studies involving human participants were reviewed and approved by the Ethical Committees of the Eye, Ear, Nose and Throat Hospital. The patients/participants provided their written informed consent to participate in this study. The animal study was reviewed and approved by the Ethical Committees of the Eye, Ear, Nose and Throat Hospital.

## Author Contributions

LZ and HC designed and conceived the experiments. JY, QH, and YG collected samples and patient details. JY, ZW, and QH completed the experiments. JY analyzed data. JY and LZ finished the paper. All authors contributed to the article and approved the submitted version.

## Funding

This study was supported by the Science and Technology Commission of Shanghai Municipality, China (Grant nos. 12J1402100 and 16411950101), the Health and Family Planning Commission of Wuxi Municipality, China (Grant no. MS201929), the Hospital Development Center (Grant no. SHDC12015114), and the Health and Family Planning Commission of Wuxi Municipality, China (Grant no. MS201645).

## Conflict of Interest

The authors declare that the research was conducted in the absence of any commercial or financial relationships that could be construed as a potential conflict of interest.

## Publisher’s Note

All claims expressed in this article are solely those of the authors and do not necessarily represent those of their affiliated organizations, or those of the publisher, the editors and the reviewers. Any product that may be evaluated in this article, or claim that may be made by its manufacturer, is not guaranteed or endorsed by the publisher.
